# Rapid Detection of Malathion, Phoxim and Thiram on Orange Surfaces Using Ag Nanoparticle Modified PDMS as Surface-Enhanced Raman Spectroscopy Substrate

**DOI:** 10.3390/foods11223597

**Published:** 2022-11-11

**Authors:** Wenlei Zhai, Mingshuo Cao, Zhiyong Xiao, Dan Li, Meng Wang

**Affiliations:** 1 Institute of Quality Standard and Testing Technology, Beijing Academy of Agriculture and Forestry Sciences, Beijing 100097, China; 2 Beijing Center of AGRI-Products Quality and Safety, Beijing 100029, China; 3 School of Chemical and Environmental Engineering, Shanghai Institute of Technology, Shanghai 201418, China

**Keywords:** surface-enhanced Raman spectroscopy, organophosphates pesticide, thiram, malathion, phoxim, Ag nanoparticles, rapid detection

## Abstract

Malathion, phoxim, and thiram are organophosphates and organosulfur pesticides widely used in agricultural products. The residues of these pesticides present a direct threat to human health. Rapid and on-site detection is critical for minimizing such risks. In this work, a simple approach was introduced using a flexible surface-enhanced Raman spectroscopy (SERS) substrate. The prepared Ag nanoparticles-polydimethylsiloxane (AgNPs-PDMS) substrate showed high SERS activity, good precision (relative standard deviation = 5.33%), and stability (30 days) after optimization. For target pesticides, the linear relationship between characteristic SERS bands and concentrations were achieved in the range of 10~1000, 100~5000, and 50~5000 μg L^−1^ with LODs down to 3.62, 41.46, and 15.69 μg L^−1^ for thiram, malathion, and phoxim, respectively. Moreover, SERS spectra of mixed samples indicated that three pesticides can be identified simultaneously, with recovery rates between 96.5 ± 3.3% and 118.9 ± 2.4%, thus providing an ideal platform for detecting more than one target. Pesticide residues on orange surfaces can be simply determined through swabbing with the flexible substrate before acquiring the SERS signal. This study demonstrated that the prepared substrate can be used for the rapid detection of pesticides on real samples. Overall, this method greatly simplified the pre-treatment procedure, thus serving as a promising analytical tool for rapid and nondestructive screening of malathion, phoxim, and thiram on various agricultural products.

## 1. Introduction

Pesticides are widely used to protect plants from insects and diseases caused by bacteria and fungi in agricultural production all over the world. Among them, organophosphate insecticides, including malathion and phoxim, can effectively kill insects by targeting their nervous system [1]. Organosulfur fungicides such as thiram are also used for the preservation of fruits and vegetables [2]. Therefore, the combination of these pesticides is commonly used in agriculture [3]. However, the overuse of pesticides has already caused serious problems in terms of environmental pollution [4]. Moreover, their residues in agricultural products pose a direct threat to human health [5,6]. For example, there are reports of several health issues caused by thiram, with symptoms such as lethargy and motor disturbances [7]. Exposure to a high enough level of malathion could lead to various symptoms, including nausea, vomiting, muscle cramps, abdominal pain, and diarrhea [8]. Phoxim may have an acute effect on the nervous systems of humans and livestock through acetylcholinesterase inhibition [9]. Typical symptoms caused by phoxim are similar to malathion, and include nausea, vomiting, diarrhea, and so forth. Because of the potential to cause those severe health issues, strict regulations have been introduced to minimize exposure to the aforementioned pesticides in many countries [10].

To better protect humans and livestock from the harmful effects of pesticides, it is urgently needed to develop effective analytical methods, especially those that can target mixed pesticides [11]. So far, malathion, phoxim, and thiram in agricultural products can be accurately measured using high-performance liquid chromatography (HPLC) [12], gas chromatography tandem mass spectrometry (GC-MS) [13], and liquid chromatography tandem mass spectrometry (LC-MS) [14]. However, these techniques require sophisticated and expensive instruments, as well as fully-trained technicians, to operate. The pre-treatment processes for food samples are also time-consuming [15]. In order to achieve rapid and on-site screening for these pesticides, many analytical methods have been developed in recent years, including electrochemical sensors [16,17], enzymatic inhibition-based sensors [18], enzyme-linked immunosorbent assay (ELISA) [19], lateral flow strip [20], surface-enhanced Raman spectroscopy (SERS) [21], microfluidics [22], and so forth. Among them, in recent decades SERS has been developed as a promising analytical platform for rapid and on-site detection of various target molecules [23,24]. Highly sensitive SERS detection depends on the enhancement of the Raman scattering signal generated from the localized surface plasmon resonance (LSPR) effect of Au or Ag nanomaterials. It presents unique advantages, including high sensitivity, instant data acquisition, highly specific molecular fingerprint from the SERS spectrum, low operating cost, and portability of devices [25,26]. Therefore, many SERS-based strategies have been reported for applications in the fields of food safety [27,28,29], environment monitoring [30], clinical diagnosis [31], security checking [32], and so forth.

In order to accomplish rapid and on-site detection of pesticides on agricultural products, a great deal of effort has been devoted to designing and fabricating Ag or Au nanomaterials as SERS substrates [33]. Recently, a new trend has been developed by combining a flexible substrate with SERS-active nanoparticles [34,35,36]. Simple sampling processes can be carried out by swabbing the target surface with the flexible substrate, while qualitative and even quantitative detection can be realized by SERS [37]. For example, a flexible SERS substrate was introduced by applying Au and Ag nanoparticles to an aluminum foil-based ZnO nanosheet. Extraction and identification of thiram on fruits and vegetables were accomplished using this substrate [38]. Apart from metal foils, polymer films have also been studied to fabricate flexible substrates for SERS [39]. Bai et al. spin-coated a monolayer of Ag island on a waterborne polyurethane emulsion film and applied the substrate for in-situ detection of thiram on apples [40]. In another study, a flexible Au nanostars/polydimethylsiloxane (PDMS) substrate was recently developed for in-situ determination of methyl parathion on fruit [41]. Wang et al. also reported the rapid detection of mixed chlorpyrifos and 2,4-dichlorophenoxyacetic acid residues on apple surfaces by using Ag colloid as a SERS substrate [42]. All these studies show the outstanding performance and great potential of applying a flexible substrate for fast and on-site SERS detection of pesticides on fruit surfaces. However, many studies still focus on a single type of pesticide, especially thiram, as the target [43,44]. New methods capable of rapidly determining mixed pesticides on one substrate are more desirable in practical applications [45].

Herein, we report the preparation of a flexible SERS substrate for the fast and simultaneous determination of three pesticides on orange surfaces. The proposed SERS substrate was fabricated by anchoring SERS-active Ag nanoparticles (AgNPs) on chemically modified PDMS thin film. The functionalization of PDMS and the surface density of AgNPs were optimized to minimize interference and achieve high SERS activity. Rhodamine 6G (R6G) was employed to evaluate the sensitivity, time stability, and signal uniformity of the prepared substrate. For pesticide detection, two organophosphate insecticides, malathion and phoxim, and the fungicide thiram were chosen as the targets. Qualitative and semi-quantitative measurement of each pesticide was established using a standard solution. Benefitting from the molecular fingerprint property of SERS spectra, the presence of each target compound can be identified according to the characteristic SERS bands, allowing simultaneous identification of the mixture of three pesticides on orange surfaces. In general, this method provides a facile and affordable platform for rapid and on-site determination of different hazardous chemicals on the surfaces of food samples.

## 2. Materials and Methods

### 2.1. Chemicals and Reagents

Silver nitrate (AgNO_3_, 99.7%) and R6G (95.0%) were purchased from Energy Chemical Co., Ltd. (Shanghai, China). Sodium citrate (99.0%) was purchased from Innochem Co., Ltd. (Beijing, China). PDMS Sylgard 184 was supplied by Dow Corning Corporation (Midland, MI, USA). (3-Aminopropyl) triethoxysilane (APTES, 99.0%) was obtained from Macklin Biochemical Co., Ltd. (Shanghai, China). Malathion (purity: 98.33%, expanded uncertainty: 0.48%), phoxim (purity: 97.26%, expanded uncertainty: 0.72%), and thiram (purity: 98.36%, expanded uncertainty: 0.56%) certified reference materials were purchased from LGC Standards GmbH (Wesel, Germany). Methanol and acetonitrile were supplied by Sinopharm Chemical Reagent Co., Ltd. (Shanghai, China). Deionized water was supplied by Titanchem Co., Ltd. (Shanghai, China) and used without further purification throughout the experiment.

### 2.2. Synthesis and Characterization of AgNPs

The AgNPs used in this study were synthesized following a modified Lee and Miesel protocol [46]. In brief, AgNO_3_ (19 mg) was added to 100 mL of deionized water. The solution was heated to boiling point. Sodium citrate solution (2 mL, 10 g L^−1^) was poured into the AgNO_3_ solution under vigorous stirring. The reaction solution was kept at gentle boiling for 30 min under continuous heating. The color of the colloid turned from light yellow to gray during the reaction. After that, the obtained Ag colloid was cooled at room temperature and stored in a refrigerator. All glassware was cleaned and rinsed with deionized water multiple times before use.

Transmission electron microscopy (TEM) images and energy-dispersive X-ray spectrum of the synthesized AgNPs were recorded with a transmission electron microscope (Tecnai G2 F30, FEI Co., Ltd., Hillsboro, OR, USA). The UV-Vis absorption spectrum was collected using a multimode plate reader (EnVision, PerkinElmer Inc., Waltham, MA, USA).

### 2.3. Preparation of Amino-Functionalized PDMS Film

The flexible PDMS film was prepared by mixing 6 g of the main agent with 0.6 g auxiliary agent. The mixture was stirred with a glass rod and poured into a 9 cm plastic petri dish to form an even and thin fluid layer. It was then transferred to a vacuum oven to remove the bubbles and heated at 60 °C for 10 h. After solidification, the transparent PDMS film was cut into small slices of squares (~5 mm × 5 mm) for further use.

For the chemical modification of the PDMS film, a series of APTES ethanol solutions were prepared with different concentrations, from 0.002% to 2% (Vf). Square slices of the PDMS films were immersed in APTES solutions for 2 h. After that, the amino-functionalized PDMS was rinsed with deionized water multiple times before drying at room temperature.

### 2.4. Preparation of AgNPs-PDMS SERS Substrate

Ag colloids with different concentrations were prepared by concentrating or diluting the as-synthesized Ag colloid. In detail, the original Ag colloid (1 mL) was pipetted into centrifuge tubes and precipitated by centrifuging at 8000 rpm for 10 min (MiniSpin, Eppendorf, Hamburg, Germany). The supernatant was carefully drawn out using a pipette, and the remaining AgNPs were resuspended with 100 μL (Ag10×), 200 μL (Ag5×), 500 μL (Ag2×), 1 mL (Ag1×), and 2 mL (Ag0.5×) deionized water, respectively.

For the fabrication of AgNPs-PDMS substrate, Ag colloid (5 μL) was dropped on the amino-functionalized PDMS film. After drying under vacuum, a uniform circle of AgNPs was formed on the PDMS. The morphology of the prepared SERS substrate was characterized using a scanning electron microscope (Quanta 650FEG, FEI Co., Ltd., Hillsboro, OR, USA).

### 2.5. Optimization and Evaluation of AgNPs-PDMS SERS Substrate

SERS activity of the prepared AgNPs-PDMS substrate was investigated by incubating with different concentrations of R6G solutions (0.1 μg L^−1^~1 mg L^−1^). SERS spectra were collected using a Raman microscope system (DXR, Thermo Fisher Scientific Inc., Waltham, MA, USA) with optimized parameters: excited at 780 nm, scanning range between 500 and 1900 cm^−1^, objective lens 10×, laser power 0.5 mW, integration time 2 s.

For evaluating signal uniformity, sixteen random spots on the substrate were chosen, and SERS spectra were collected after incubating with 100 μg L^−1^ R6G solution. Time stability was also examined by collecting the SERS spectrum every 5 days. The tested substrate was stored in a sealed tube filled with nitrogen gas after each measurement.

### 2.6. Semi-Quantitative Analysis of Three Pesticides and Determination of Intra-Batch and Inter-batch Precision

Firstly, malathion and phoxim stork solutions with a concentration of 1000 mg L^−1^ were prepared by dissolving 1 μL of malathion and phoxim in 1.230 mL and 1.176 mL acetonitrile, respectively. Standard solutions with sixteen different concentrations (100, 50, 20, 10, 5, 2, 1 mg L^−1^ and 500, 200, 100, 50, 20, 10, 5, 2, 1 μg L^−1^) were prepared by diluting the stork solution with corresponding amounts of solvent. Similarly, thiram stock solution was prepared by first weighing 10 mg thiram, dissolved in 10 mL methanol and diluted into solutions with sixteen different concentrations. Mixed solutions were prepared by mixing three standard solutions at different concentrations.

For semi-quantitative analysis of the three pesticides, SERS spectra of different concentrations of standard solutions were acquired with the following parameter setting: laser power 1 mW, integration time 5 s. In order to evaluate the intra-batch precision of this method, AgNPs-PDMS substrates fabricated in the same batch were used to record SERS spectra of 1000 μg L^−1^ thiram, malathion, and phoxim, respectively. Six SERS spectra were collected for each pesticide, and the relative standard deviation (RSD) of SERS intensity was calculated as the intra-batch precision. For inter-batch precision, six batches of AgNPs-PDMS substrates were prepared, then SERS spectra were recorded for each pesticide at the concentration of 1000 μg L^−1^. The inter-batch precision was determined by calculation of the RSD of SERS signals generated from different batches of AgNPs-PDMS substrates.

### 2.7. Detection of Three Pesticides on Orange Surfaces

Thiram, malathion, and phoxim were tested on the surfaces of oranges from a local market to examine the performance of the AgNPs-PDMS substrate. Bought oranges were washed with water to clean the surface. Then, 10 μL mixed solution of pesticides at different concentrations (100 μg L^−1^ for thiram, 500 μg L^−1^ for malathion, 200 μg L^−1^ for phoxim) was spiked on the orange peel. After solvent evaporation, the spot was sprayed with a small amount of ethanol, pressed, and gently swabbed by the AgNPs-PDMS film to transfer pesticide residues onto the substrate. The SERS signal of the swabbed substrate was acquired for qualitative and semi-quantitative analysis. The Raman parameter setting was as follows: excitation wavelength and scanning range remained the same as before, laser power 3 mW, integration time 10 s.

### 2.8. Statistical Analysis

In this study, each sample was scanned three times, and the average signal was recorded as the SERS spectra. Baseline correction was performed using OMNIC 9 software (Thermo Fisher scientific Inc., Waltham, MA, USA). After that, spectral data were implemented in OriginPro 8 software (OriginLab Corporation, Northampton, MA, USA) for further analysis, including integration of the peak areas, linear fitting to obtain the regression equations, and calculation of relative standard deviation (RSD). The size distribution of the synthesized AgNPs was counted using ImageJ software (National Institutes of Health, Bethesda, MD, USA). All figures were plotted using OriginPro 8 software.

## 3. Results and Discussion

### 3.1. Characterization of the Synthesized AgNPs

**Figure 1:**
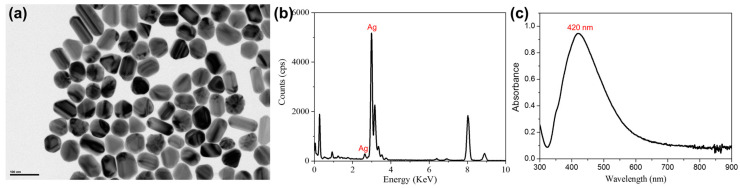
TEM image (**a**), energy-dispersive X-ray spectrum (**b**), and UV-Vis absorption spectrum (**c**) of the synthesized AgNPs. Scale bar in **a**: 100 nm.

In this study, TEM images, energy-dispersive X-ray, and UV-Vis spectroscopy were first used to characterize the synthesized AgNPs. As shown in Figure 1a, the AgNPs were well dispersed and the average size was measured as 70~80 nm. The energy-dispersive X-ray spectrum presented in Figure 1b confirmed the synthesized nanoparticles were composed of Ag. The UV-Vis absorption spectrum (see Figure 1b) showed a characteristic peak of AgNPs at 420 nm, which agreed well with previous literature reports [47]. The relatively narrow half band width of the absorption curve suggested the synthesized AgNPs had good size distribution [48].

**Figure 2:**
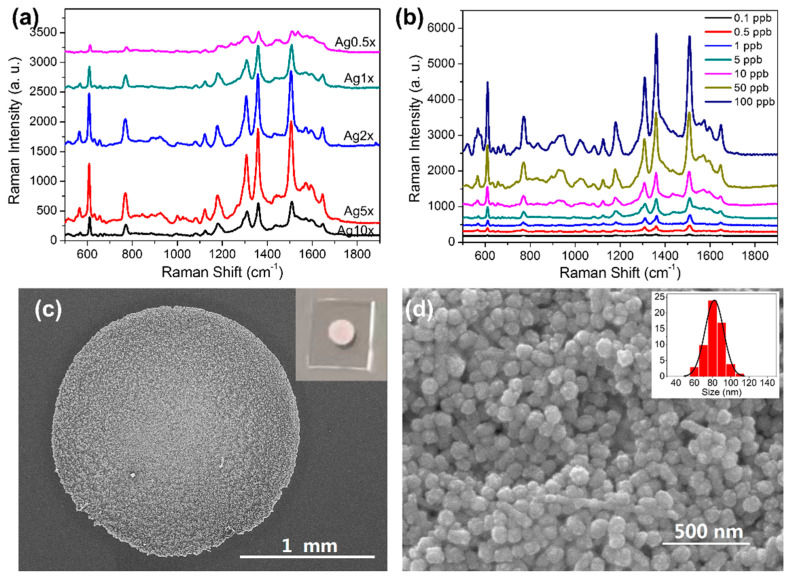
SERS spectra of R6G on AgNPs-PDMS substrates with various surface densities of AgNPs (**a**). SERS spectra of R6G (0.1~100 μg L^−1^) on optimized AgNPs-PDMS substrate (**b**). SEM images of the optimized AgNPs-PDMS substrate under different scales (**c**,**d**). Inset of **c**: photo of the prepared substrate. Inset of **d**: statistical analysis of size distribution of the modified AgNPs.

The surface of PDMS film exhibits a hydrophobic effect, making it undesirable for coating with aqueous Ag colloid. Therefore, chemical modification was carried out by simply incubating the PDMS film in an APTES ethanol solution. Presumably, a small number of hydroxyl groups on the PDMS surface enabled the formation of chemical bonds with APTES, resulting in amino functionalization of PDMS and reversing the hydrophobic property. After dropping 5 μL concentrated Ag colloid and drying in a vacuum, a small circle with metallic appearance was observed, as presented in the inset of Figure 2c and in Figure S1.

### 3.2. Optimization and Characterization of AgNPs-PDMS Substrate

As the first step of modification, the amino functionalization process was optimized by adjusting the concentration of the APTES solution. PDMS slices were immersed in different concentrations of APTES ethanol solutions (0.002%, 0.005%, 0.01%, 0.02%, 0.05%, 0.1%, 0.2%, 0.5%, 1%, and 2%, Vf). After dropping Ag colloid and drying, it was observed that the minimum concentration of APTES was 0.01% for the successful formation of the circular AgNPs substrate (see Supplementary Materials, Figure S1), and it was established as the optimized condition for further experiments. Notably, by using a higher amount of APTES, the obtained AgNPs substrate was encapsulated with a thin layer of transparent coating in the SEM image (see Supplementary Materials, Figure S2), presumably caused by the self-polymerization of extra APTES [49]. In the SERS measurements, this coating material could prevent the access of target molecules to the proximity of AgNPs, resulting in poor sensitivity. Therefore, it is necessary to restrict the use of APTES to a minimum required level. Compared with a previous report, which required the treatment of PDMS with piranha solution before functionalization [41], this method simplifies the modification process and avoids the preparation and use of dangerous reagents.

After amino functionalization, the amount of AgNPs is another crucial factor to determine SERS activity. Thus, Ag colloids with different concentrations were prepared and evaluated. R6G has been extensively used as a probe molecule for SERS due to its excellent Raman properties [50]. In this study, it was also employed to investigate and optimize the performance of our substrate. As presented in Figure 2a, the SERS spectra of 100 μg L^−1^ R6G solution showed that PDMS modified with Ag5×, which was 5-fold concentrated from the original Ag colloid, produced the strongest SERS signal. The peak intensity at 1358 cm^−1^ was five times higher than Ag0.5×. This might be caused by the fully packed AgNPs through the concentrating process, which generate more “hot spot” nanostructures in SERS enhancement. The enhancement factor (EF) for R6G was calculated based on the widely adopted equation [51]:

$$EF=\frac{I_{SERS}/I_{bulk}}{N_{SERS}/N_{bulk}}$$

where *I_SERS_* and *I_bulk_* stand for peak intensities at 1358 cm^−1^ from both SERS and Raman spectrum of R6G, respectively. *N_SERS_* and *N_bulk_* represent the estimated number of molecules stroked by the laser on the SERS substrate and R6G powder. The calculation process is detailed in Supplementary Materials, Figure S3. As a result, the EF of the optimized substrate was estimated to be 1.64 × 10^5^, which is sufficient to provide significant enhancement of Raman intensity from analytes adsorbed on the AgNPs-PDMS substrate [52].

In Figure 2b, SERS spectra of R6G with different concentrations are presented. The SERS intensity gradually increased with higher concentration of the tested solutions. As low as 0.1 μg L^−1^ of R6G can be detected using the AgNPs-PDMS substrate. This result indicates it is feasible to use this substrate for semi-quantitative analysis.

After optimization, the morphology of the optimized substrate was characterized via scanning electron microscopy (SEM). From the image shown in Figure 2c, we can observe that the diameter of the circle was approximately 2 mm. The zoomed in image shows AgNPs were densely distributed on the PDMS film (see Figure 2d). The approximate distance between AgNPs could lead to strong SERS enhancement due to the generation of “hot spots” for molecules trapped between the gaps of AgNPs [53]. Moreover, the relatively even distribution of aggregated AgNPs guarantees a uniform distribution of the SERS signal, which is also vital for reliable SERS measurement. Statistical analysis of the distribution of particle size is presented in the inset of Figure 2d. The result showed AgNPs with sizes between 80 and 90 nm are dominant, which agreed well with the observation of TEM image and UV-Vis spectrum.

### 3.3. Evaluation of Signal Uniformity and Stability

**Figure 3:**
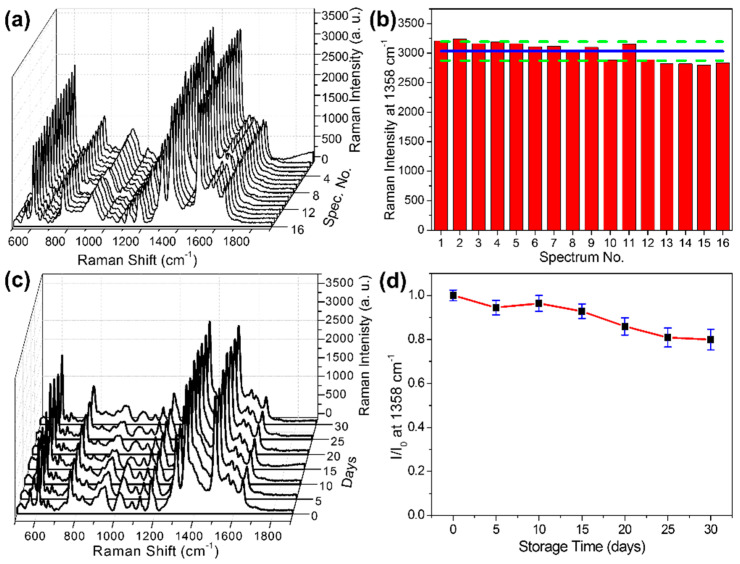
SERS spectra (**a**) and distribution of signal intensity (**b**) at 1358 cm^−1^ of R6G (100 μg L^−1^) collected from 16 random points on optimized AgNPs−PDMS substrate. The solid line in b represents the average SERS intensity, and the dotted lines indicate the RSD. SERS spectra (**c**) and the ratio of intensities (**d**) at 1358 cm^−1^ of R6G (100 μg L^−1^) acquired on optimized AgNPs-PDMS sub-strate stored in nitrogen atmosphere between 0 and 30 days.

Apart from sensitivity, signal uniformity is another important aspect for assessing the performance of SERS substrates. In this experiment, 100 μg L^−1^ R6G was still used as the testing solution. As shown in Figure 3a, by measuring 16 randomly distributed points on the optimized substrate, signal intensity at 1358 cm^−1^ was analyzed, and the result showed the prepared substrate provided uniform enhancement of the Raman signal. The RSD was calculated as 5.33% (see Figure 3b), which is comparable with previous studies. In practical application, a SERS substrate with longer time stability has extra advantage. To evaluate the stability of our AgNPs-PDMS substrate, 100 μg L^−1^ R6G solution was dropped and dried. After recording the SERS spectrum, it was stored in a nitrogen environment and remeasured every 5 days. From Figure 3c,d, we can tell that the substrate showed relatively good stability, with 80% SERS activity preserved in the period of one month. Even though a decrease of SERS activity was observed, considering the easy to oxidize nature of Ag, the stability of this substrate is still acceptable [54]. In addition, benefitting from the easy to fabricate procedure and low cost, it is easy to resupply the substrate in large quantities.

### 3.4. Detection of Individual Pesticide on AgNPs-PDMS Substrate

**Figure 4:**
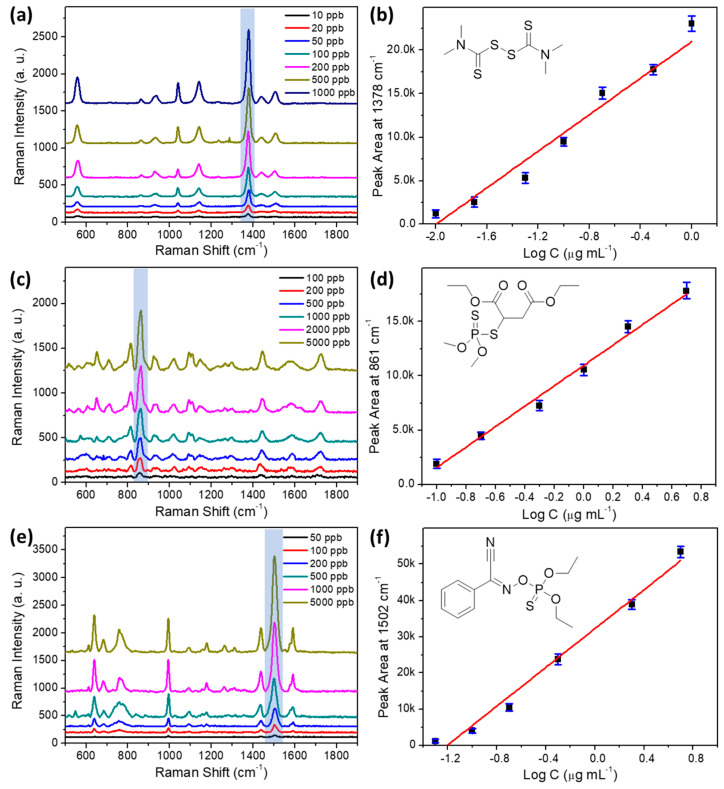
SERS spectra of different concentrations of thiram (**a**), malathion (**c**), and phoxim (**e**). Linear calibration plots between the integrated peak area and the logarithm of concentration for thiram (**b**), malathion (**d**), and phoxim (**f**). Inset of (**b**,**d**,**f**): molecular structure of thiram, malathion, and phoxim.

The optimized substrate was employed to test standard solutions of thiram, malathion, and phoxim. Figure 4 presents the SERS spectra of different concentrations of thiram (a), malathion (c), and phoxim (e). The linear calibration plots of all three pesticides are also illustrated in Figure 4b,d,f, respectively. SERS detection of thiram has been extensively studied in previous reports [38,40,43,44]. In this study, thiram was chosen first for pesticide detection. As illustrated in Figure 4a, characteristic peaks at 558, 925, 1147, 1384, and 1513 cm^−1^ are identified and assigned in Table 1 according to Figure S4a and the literature [55]. In detail, the SERS band at 558 cm^−1^ corresponds to the stretching of the S−S bond. The broad peak at 925 cm^−1^ is the combination of the stretching mode of the C=S double bond and CH_3_N. The major signal at 1384 cm^−1^ is assigned to symmetric CH_3_ deformation. The Raman shift of all these peaks matches well with previous literature reports. Among them, the peak at 1384 cm^−1^ provides the highest SERS intensity, thus it was chosen for semi-quantitative analysis. As presented in Figure 4b, for thiram between the concentrations of 10 and 1000 μg L^−1^, the plotting of an integrated peak area at 1384 cm^−1^ against the logarithm of thiram concentrations presents a linear relationship, with a coefficient of determination (R^2^) of 0.9665. This result demonstrates that the proposed substrate can be applied for qualitative and semi-quantitative detection of thiram at low levels. The limit of detection (LOD) is calculated as 3.62 μg L^−1^ using the widely adopted formula LOD = 3σ/S, in which σ represents the standard deviation of background signal and S stands for the slope of the linear calibration curve. For practical application, the maximum residue limit (MRL) for thiram in apple is 5 mg kg^−1^, according to the national food safety standard (GB 2763-2019, China). Therefore, the sensitivity of this method is satisfied for thiram detection.

For organophosphates insecticides, malathion is tested first. The resulted SERS spectra are presented in Figure 4c. By comparing with the Raman spectrum of the reference material, it is confirmed that the recorded SERS signal is produced by SERS enhancement of malathion molecules (see Supplementary Materials, Figure S4b). The characteristic Raman band at 815 cm^−1^ is attributed to the out-of-plane bending of the C−H bond. The strong signal at 862 cm^−1^ is caused by the symmetric stretching mode of the C−O−C bond. The broad peak at 1144 cm^−1^ is generated from the stretching of the P−S bond. Finally, the two bands at 1444 and 1726 cm^−1^ are correlated with the stretching mode of the C−O and C=O bonds, respectively. The pattern of these characteristic peaks can be used for qualitative identification of malathion. For semi-quantitative detection, Figure 4d shows the linear fitting curve of data plotted between the logarithm of malathion concentration and integrated peak area at 862 cm^−1^. As summarized in Table 2, a linear range of 100 to 5000 μg L^−1^ is achieved using this method, with a calculated LOD of 41.46 μg L^−1^. Even though the sensitivity is not as high as thiram, probably due to the weak affinity between malathion molecules and the Ag substrate, it can still be useful for on-site inspection, considering the relatively high MRL of 4 mg kg^−1^ in oranges.

Phoxim is another target insecticide in this study due to its known toxicity, which is an acetylcholinesterase inhibitor that affects the human nervous system. According to GB 2763-2019, the MRLs of phoxim are set as 0.05 mg kg^−1^ in most fruits. Unlike thiram, SERS detection of phoxim has rarely been reported. Using an optimized AgNPs-PDMS substrate, SERS measurement of different concentrations of phoxim standard solutions were performed. Characteristic peaks can be observed on the acquired SERS spectra in Figure 4e. By comparing with the Raman spectrum of phoxim in Figure S4c, characteristic SERS bands can be identified. Among them, the broad peak at 758 cm^−1^ should be recognized as the stretching vibration of the P=S double bond. Peaks at 995, 1092, and 1178 cm^−1^ are all attributed to the in-plane bending of the C−H bond. Different from thiram and malathion, SERS spectra of phoxim showed characteristic stretching vibration signals of the phenyl ring at 1437, 1502, and 1591 cm^−1^, which provide important information for distinguishing its signal in a mixed sample. For semi-quantitative detection, the strongest peak at 1502 cm^−1^ is used as a reference. A linear relationship is established between the logarithm of concentrations and signal strength. The linear range is between 50 and 5000 μg L^−1^, with LOD reaching 15.69 μg L^−1^.

As presented in Table 2, the R^2^ of the calibration curves are in the range of 0.9665~0.9891 for the tested pesticides. Comparing with other quantitative analysis techniques such as GC-MS and LC-MS, the linearity of this method is relatively poorer. It might be caused by surface defects on the AgNPs-PDMS substrate, which give rise to uneven enhancement of the Raman signal. Nonetheless, the coefficients of determination achieved in this study are at the same level with previously reported SERS-based methods [41,42,56]. Considering that the priority of this study is to establish a method for the rapid screening of pesticide residues, the linearity of the regression equations should be acceptable for semi-quantitative SERS analysis.

To investigate the precision of this method for pesticide detection, a series of experiments was conducted by measuring the SERS spectra of each pesticide using substrates from the same and different batches. The results are summarized in Table 2. For the tested pesticides, the RSDs are between 3.18% and 4.65% for the SERS signals obtained from the same batch of AgNPs-PDMS substrates. In the case of inter-batch precision, SERS spectra from six different batches of substrates were collected, and the RSDs varies from 5.96% to 8.27%. These results indicate that consistent SERS measurements can be performed on different substrates, and the precision of this method is acceptable for rapid and on-site detection.

### 3.5. Detection of Mixed Pesticides on Orange Surface Using AgNPs-PDMS Substrate

**Figure 5:**
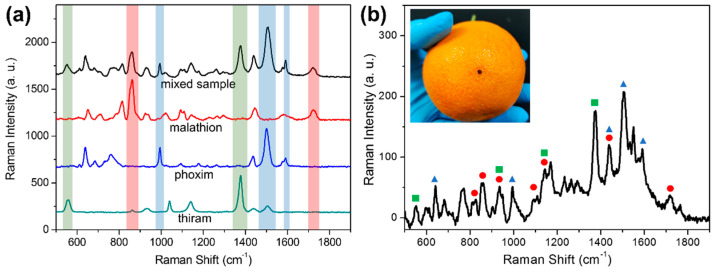
SERS spectra of mixed (black curve) and single pesticides (thiram, malathion, and phoxim) using an AgNPs-PDMS substrate (**a**). SERS spectrum of orange surface spiked with mixed pesticides after swab sampling using an AgNPs−PDMS substrate; characteristic SERS bands are labeled for each pesticide: thiram (green square), malathion (red circle), and phoxim (blue triangle) (**b**). Inset of (**b**): photo of performing swab sampling using the flexible SERS substrate.

After determining the calibration curve of each pesticide using the AgNPs-PDMS substrate, our next aim is to accomplish semi-quantitative detection of three pesticides simultaneously. A mixed solution of malathion (500 μg L^−1^), thiram (100 μg L^−1^), and phoxim (200 μg L^−1^) was prepared and measured by SERS using the AgNPs-PDMS substrate. In Figure 5a, the characteristic peaks in the SERS spectrum of the mixed sample can be used for distinguishing each pesticide. As labeled in different colors, for each component there are at least two or three exclusive peaks without overlapping. In other words, benefiting from the molecular fingerprint property of SERS, the characteristic information of each pesticide can be extracted from the SERS signal of the mixed sample. According to integrated peak areas at 860, 1378, and 1506 cm^−1^, the recovery rates were calculated as 104.6 ± 1.9%, 96.5 ± 3.3%, and 118.9 ± 2.4% for malathion, thiram, and phoxim, respectively. The recovery rate for phoxim is higher than expected, presumably due to slightly overlapping with the weak band of thiram at 1513 cm^−1^. Nonetheless, this study confirms that the semi-quantitative analysis of three pesticides from a complex environment is feasible using this method.

Benefitting from the flexible property of the AgNPs-PDMS substrate, it is possible to transfer pesticide residues from the orange surface onto the substrate for SERS measurement. Comparing with other techniques, this method does not require the additional pre-treatment of oranges samples. In this work, a mixed solution of three pesticides was spiked onto the surface of oranges as the tested sample. After solvent evaporation, the orange surface was sprayed with the minimum amount of ethanol and covered by the AgNPs-PDMS substrate. As shown in the inset of Figure 5b, through gentle pressing and swabbing, adequate contact between the orange surface and the substrate was made, thus sampling of the spiked pesticides was completed. From the SERS spectrum presented in Figure 5b, it is obvious that the characteristic peaks of each pesticide can be identified. In detail, SERS signals at 552 and 1376 cm^−1^ are attributed to thiram; peaks at 811, 860, 1093, and 1718 cm^−1^ are assigned to malathion; and peaks at 640, 995, 1506, and 1591 cm^−1^ indicate the presence of phoxim. It is worth noting that no obvious matrix interferences can be observed in the SERS spectrum. Presumably, the surface of the tested orange did not break down owing to the swab sampling approach, resulting in an almost neglectable matrix effect for SERS measurements. This experiment demonstrates that it is feasible to conduct the rapid screening of mixed pesticides on orange surfaces using this flexible and low-cost SERS substrate.

## 4. Conclusions

In summary, a simple and robust method was proposed for the rapid screening of three pesticide residues on orange surfaces using SERS. Flexible SERS substrates were prepared by modifying AgNPs on amino-functionalized PDMS films. The optimized substrates showed high sensitivity, good uniformity, and stability. For pesticide detection, thiram, malathion, and phoxim were measured using this substrate. Linear relationships were established for each analyte, enabling semi-quantitative detection of these pesticides by SERS. Moreover, the simultaneous determination of three pesticides on orange surfaces was achieved by performing a swab sampling process. Compared with other methods, this approach allows rapid and nondestructive detection of mixed pesticides, with low-cost material and an easy to operate procedure. No extra treatment of samples is required before testing, and the substrate can be directly discarded after use. It is feasible to further extend this method for the rapid and on-site detection of other hazardous substances on various fruits.

## Figures and Tables

**Table 1:** Assignments of some characteristic bands in Raman and SERS spectra of three tested pesticides.

Analyte	Raman/cm^−1^	SERS/cm^−1^	Assignments
thiram	557 973	558 (m) 925 (w)	*ν*(S−S) *ν*(CH_3_N); *ν*(C=S)
	1149	1147 (m)	*ρ*(CH_3_); *ν*(C−N)
	1374 1396 1455	1384 (s) 1443 (w) 1513 (m)	*δ*_s_(CH_3_); *ν*(C−N) *δ*_as_(CH_3_) *ν*(C−N); *δ*(CH_3_); *ρ*(CH_3_)
malathion	716 824 858 1096	709 (b, w) 815 (m) 862 (s) 1094 (m)	*ν*(P=S); *ν*(P−O) *γ*(C−H) *ν*_s_(C−O−C) *ν*(C−C)
	1164 1452 1736	1144 (w) 1444 (m) 1726 (m)	*ν*(P−S) *ν*_s_(C−O) *ν*(C=C)
phoxim	750 999	758 (b, m) 995 (m)	*ν*(P=S) *γ*(C−H)
	1098 1182	1092 (w) 1178 (w)	*γ*(C−H) *γ*(C−H)
	1302 1446 1555 1597	1264 (w) 1437 (m) 1502 (s) 1591 (m)	*δ*_s_(CH_3_) *ν*_as_(CH_3_) *ν*(C_6_H_5_) *ν*(C_6_H_5_)

b—broad; s—strong; m—middle; w—weak; *ν*—stretching; *δ*—bending; *ρ*—rocking; *γ*—out-of-plane bending; *ν*_s_—symmetric stretching; *ν*_as_—asymmetric stretching; *δ*_s_—symmetric bending; *δ*_as_—asymmetric bending.

**Table 2:** Summary of validation parameters of the proposed method.

Parameters	Thiram	Malathion	Phoxim
Characteristic peak for semi-quantitative analysis (cm^−1^)	1378	861	1502
LOD (μg L^−1^)	3.62	41.46	15.69
Linear range (μg L^−1^)	10~1000	100~5000	50~5000
Regression equation	y = 11,226.81x + 21,852.76	y = 9556.87x + 11,017.77	y = 26,790.12x + 32,245.99
R^2^	0.9665	0.9891	0.9805
Intra-batch precision (% RSD)	4.65	3.74	3.18
Inter-batch precision (% RSD)	8.27	5.96	7.03
Recovery rate (%) *	96.5 ± 3.3	104.6 ± 1.9	118.9 ± 2.4

* Recovery rates were calculated according to SERS spectra of the mixed samples.

## Data Availability

Data is contained within the article or Supplementary Materials.

## Supplementary Materials

The following supporting information can be downloaded at: https://www.mdpi.com/article/10.3390/foods11223597/s1, Figure S1: Optical images of AgNPs-PDMS substrates prepared with different concentrations (0.002~0.5%) of APTES. Figure S2: SEM images of AgNPs-PDMS substrates prepared with 0.01% (a) and 2% (b) APTES modified PDMS. Figure S3: SERS spectrum of 100 μg L^−1^ R6G on AgNPs-PDMS substrate (red curve); and Raman spectrum of R6G powder acquired under the same condition (black curve). Figure S4: Normalized SERS (red curve) and Raman (black curve) spectra of three tested pesticides: thiram (a), malathion (b), and phoxim (c) [49,50].
