# Supplementary material for: Rapid Detection of Malathion, Phoxim and Thiram on Orange Surfaces Using Ag Nanoparticle Modified PDMS as Surface-Enhanced Raman Spectroscopy Substrate
Source: Foods. 2022 Nov 11;11(22):3597. doi: 10.3390/foods11223597 (PMC9689543; doi:10.3390/foods11223597)
Supplement: Supplementary file 1 [file foods-11-03597-s001.zip › foods-1981115-supplementary.pdf]

Rapid Detection of Malathion, Phoximand Thiram on Orange Surfaces Using Ag Nanoparticle Modified PDMS as Surface-Enhanced Raman Spectroscopy Substrate

(Supplementary Material)

Wenlei Zhai [1], Mingshuo Cao [1], Zhiyong Xiao [2], Dan Li [3] and Meng Wang [1,*]

1 Institute of Quality Standard and Testing Technology, Beijing Academy of Agriculture and Forestry Sciences, Beijing 100097, China; zhaiwl@iqstt.cn (W. Z.); 17832071720@163.com (M. C.)

2 Beijing Center of AGRI-Products Quality and Safety, Beijing 100029, China; hjzncp@126.com

3 School of Chemical and Environmental Engineering, Shanghai Institute of Technology, Shanghai 201418, China; dany@sit.edu.cn

* Correspondence: wangm@iqstt.cn

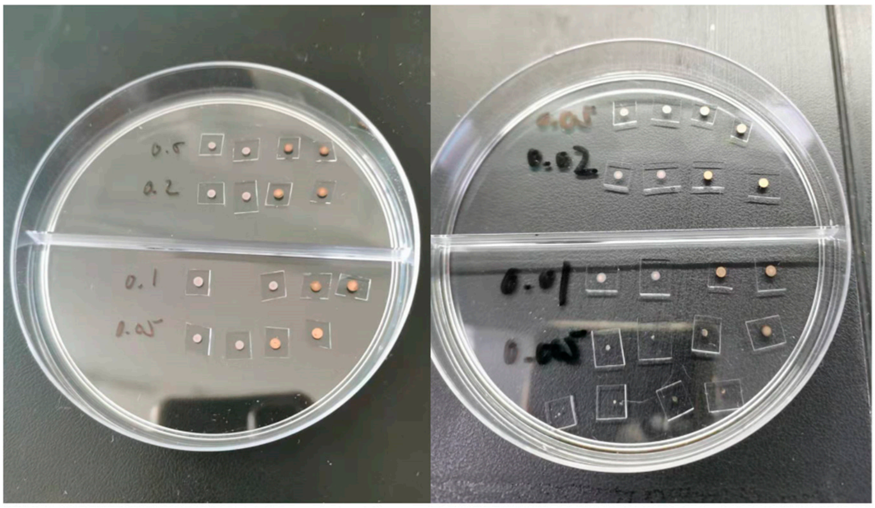

Figure S1. Optical images of AgNPs-PDMS substrates prepared with different concentrations (0.002% ~ 0.5%) of APTES. Two parallel samples were prepared for each condition. Also, both AgNPs (pale gray) and AuNPs (yellow) were used for modification.

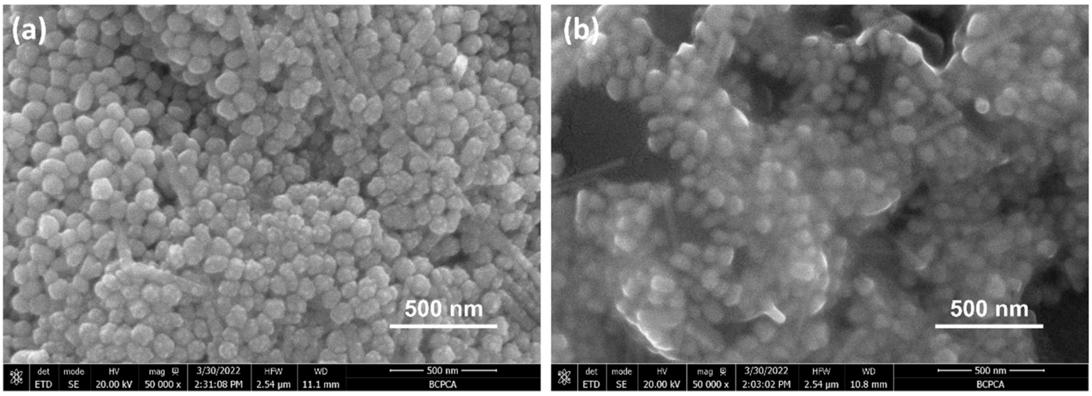

Figure S2. SEM images of AgNPs-PDMS substrates prepared with 0.01% (a) and 2% (b) APTES modified PDMS.

Calculation of enhancement factor (EF) of the optimized SERS substrate:

EF has been widely used for characterization of SERS activity of the fabricated substrate. In this work, R6G was selected as the probe molecule. EF of the optimized AgNPs-PDMS substrate for R6G was calculated following the widely adopted method in literature reports [1,2]:

$$EF = \frac{I_{SERS}/I_{bulk}}{N_{SERS}/N_{bulk}}$$

where I_{SERS} and I_{bulk} are the peak intensities at 1358 cm^{-1} of SERS and normal Raman spectra of R6G, respectively. N_{SERS} and N_{bulk} are the estimated number of molecules under laser excitation for the estimated number of molecules adsorbed on SERS substrate and the bulk sample, respectively. We assumed the R6G molecules distributed evenly on SERS substrate. Therefore, N_{SERS} can be estimated according to the average surface density of R6G and the area of the laser spot. In this experiment, 2.5 µL of 100 ng mL^{-1} (2.26×10^{-7} mol L^{-1}) R6G solution was pipetted on the substrate. After drying, a circular spot with the diameter of 1.80 mm was formed. Thus the average surface density of R6G was estimated to be 2.22×10^{-19} mol μm^{-2}. The area of the laser spot was calculated to be 7.07 μm^2 according to the diameter of the laser beam (ca. 3 µm). Therefore, the estimated N_{SERS} has a value of 1.57×10^{-18} mol. For the bulk sample of R6G, the sampling volume depends on the area and penetration depth (ca. 4 µm) of the laser beam. Taking the density of bulk R6G (1.15 g cm^{-3}) into account, N_{bulk} is calculated to be 7.36×10^{-14} mol. For signal intensity at 1358 cm^{-1}, the ratio of I_{SERS} and I_{bulk} is approximately 3.5 : 1 (see Figure S1). As a result, the EF value of the substrate for R6G was estimated to be 1.64×10^5.

Adapted with permissions from Ref. [1, 2]. 2012, the Royal Society of Chemistry.

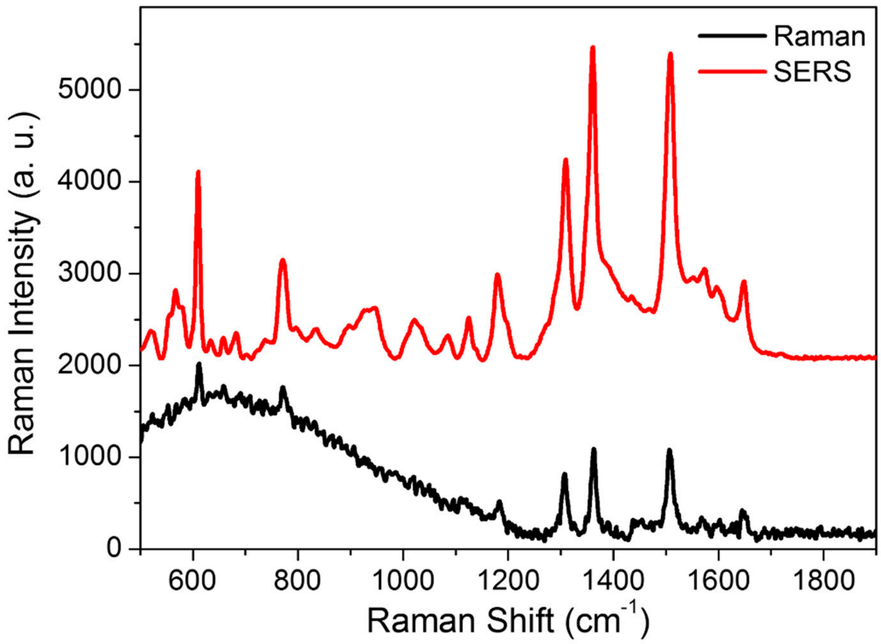

Figure S3. SERS spectrum of 100 μg L^{-1} R6G on AgNPs-PDMS substrate (red curve); and Raman spectrum of R6G powder acquired under the same condition (black curve).

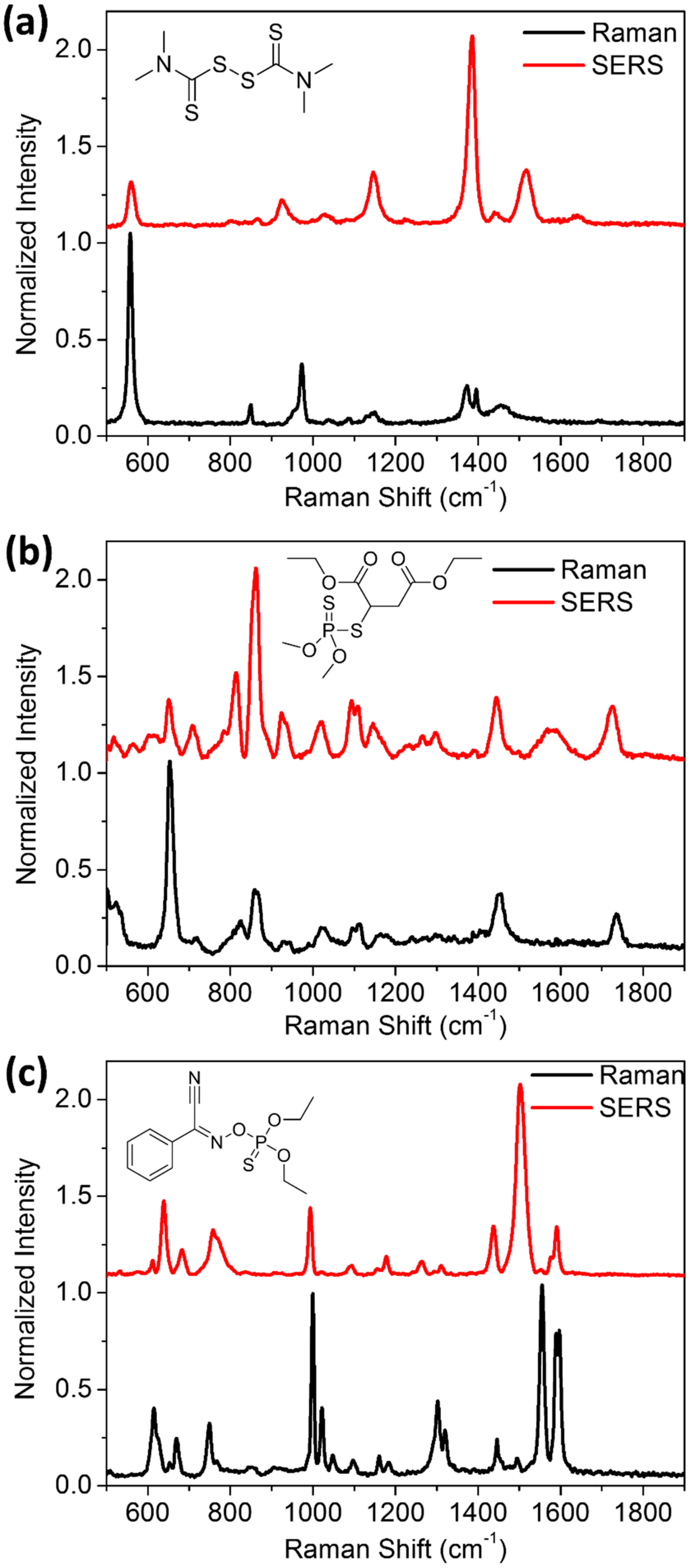

Figure S4. Normalized SERS (red curve) and Raman (black curve) spectra of three tested pesticides: thiram (a), malathion (b) and phoxim (c).

Reference:

1. Zhai, W.-L.; Li, D.-W.; Qu, L.-L.; Fossey, J.S.; Long, Y.-T. Multiple depositions of Ag nanoparticles on chemically modified agarose films for surface-enhanced Raman spectroscopy. *Nanoscale* **2012**, *4*, 137-142, doi:10.1039/C1NR10956A.
2. Qu, L.-L.; Li, D.-W.; Xue, J.-Q.; Zhai, W.-L.; Fossey, J.S.; Long, Y.-T. Batch fabrication of disposable screen printed SERS arrays. *Lab Chip* **2012**, *12*, 876-881, doi:10.1039/C2LC20926H.
